# The role of neighborhood inequalities on diabetes prevention care: a mini-review

**DOI:** 10.3389/fcdhc.2023.1292006

**Published:** 2023-11-15

**Authors:** Francesco Frigerio, Luca Muzzioli, Alessandro Pinto, Lorenzo Maria Donini, Eleonora Poggiogalle

**Affiliations:** Department of Experimental Medicine, Sapienza University of Rome, Rome, Italy

**Keywords:** diabetes, T2DM, inequalities, social determinants of health, neighborhood, residence characteristics

## Abstract

An emerging research niche has focused on the link between social determinants of health and diabetes mellitus, one of the most prevalent non-communicable diseases in modern society. The aim of the present mini-review is to explore and summarize current findings in this field targeting high-income countries. In the presence of disadvantaged neighborhood factors (including socioeconomic status, food environment, walkability and neighborhood aesthetics), diabetes prevention and care are affected at a multidimensional level. The vast majority of the included studies suggest that, besides individual risk factors, aggregated neighborhood inequalities should be tackled to implement effective evidence-based policies for diabetes mellitus.

## Introduction

According to the most recent global data, from 1990 to 2021 the estimated annual percentage in diabetes mellitus prevalence rose from 3.2% to 6.1%. Between-country analysis revealed that a rise in diabetes incidence and prevalence has occurred globally, independent of country-level income (1). A relevant research niche, focusing on neighborhood disparities, has been growing in interest with interventional studies showing encouraging results in terms of diabetes prevalence reduction (2). Besides disease prevention, a number of domains of diabetes care, from glycemic control to complications and economic burden, may be affected by social determinants of health at the residential level. The aim of the present review is to assess to which extent neighborhood factors are associated with diabetes mellitus.

## Methods

We searched the Pubmed/MEDLINE database for relevant studies published in the last 10 years. Our search strategy combined Medical subject heading (MeSH) terms (“Diabetes mellitus”, “social determinants of health”, “Residence Characteristics”) with relevant keywords (diabetes, neighborhood, neighbourhood, income, "economic status", social, old, aged, handgrip, grip, strength) and truncated words (function*, dynap*, sarcop*, frail*, elder*) connected by Boolean operators (AND, OR, NOT). Exclusion criteria were: low- or middle-income countries, languages other than English. The latest search was performed on August 3, 2023. The search retrieved 2177 records; after screening by title and abstract, 49 reports were searched for retrieval, of which 33 articles were selected. Furthermore, 2 additional reports were obtained through cross-citation, leading to the final inclusion of 35 articles in the present study. Since our search was limited to one database, included only high-income countries and aimed to present a brief state-of-the-art summary on the topic, the mini-review format was adopted.

## Neighborhood characteristics influencing diabetes incidence and prevalence

### Neighborhood socioeconomic disadvantage

Several neighborhood aspects have been identified and their association with the risk of diabetes has been explored. Among these factors, socioeconomic disadvantage is the most studied. In Bilal et al. (3) 199,621 residents (mean age: 57.6 years; 56% women) of Spanish neighborhoods undergoing greater transformation processes (e.g., new housing and improved socioeconomic status, SES) showed a lower hazard ratio (HR) of diabetes risk than people living in stable areas. In another study, neighborhoods with a high level of disadvantage showed an increased risk of obesity and diabetes, as well as higher odds ratios (ORs) for hypertension and fatty liver (4). Furthermore, in a longitudinal study on 11,035 Australians self-reporting a set of NCDs (min-max age: 40-65 years; 55% women), residents of disadvantaged neighborhoods showed higher ORs of Type 2 Diabetes Mellitus (T2DM) onset (OR: 2.21), heart disease (OR: 1.72), and comorbidity (OR: 4.38) compared to districts with higher socioeconomic status (5). In an elegant Swedish study, 61,386 refugees (min-max age: 25-50 years; 53% women) were randomly assigned to different SES neighborhoods at their entrance into the country; high-deprivation areas increased the refugees’ risk of diabetes and prolonged exposure to high-deprivation neighborhoods (up to 5 years) was associated with a 9% increase in diabetes risk (6). Similar findings emerged from a US study, revealing a higher diabetes prevalence (24.5%) in urban settings compared to either suburban or small-town areas (18.5%), suggesting that individual lifestyle and other neighborhood attributes could also affect diabetes prevalence in high-density residential areas (7). Moreover, the rate of unemployment was associated with an increased risk of T2DM (HR: 1.72) in a German cohort (n = 7,250; min-max age: 45-74 years; 51% women), regardless of any individual characteristic (8).

### Neighborhood environmental attributes: walkability, green space and air pollution

Walkability is a physical environmental characteristic of neighborhood and urban design, which has the potential to influence different types of physical activity, such as walking and cycling. Neighborhood walkability is composed of three factors: street connectivity, residential density, and land use mix (9). A Canadian longitudinal study on 958,567 adults (age > 18 years) found that neighborhoods with comparable walkability levels, even when located in different regions, showed similar diabetes incidence rates, regardless of immigration status and income level. Neighborhoods with high walkability were associated with a lower incidence of diabetes among adults (8.2 vs. 9.2 per 1000; HR: 0.85) but not among the elders (20.7 vs. 19.5 per 1000; HR: 1.01) (10). Another Canadian study analyzed 8,777 neighborhoods across 7 cities with a Walkability Index ranging from 10.1 to 35.2. Over a 12-year period, areas with low walkability showed an increased prevalence of overweight and obesity (absolute changes: 5.4% in quintile 1, 6.7% in quintile 2 and 9.2% in quintile 3). On the other hand, high walkability areas displayed a reduction in diabetes incidence: from 7.7 to 6.2 per 1000 persons in quintile 5 (absolute change: −1.5) and from 8.7 to 7.6 in quintile 4 (absolute change: −1.1) (11). Nonetheless, a Swedish study found an inverse association between T2DM and neighborhood walkability, but statistical significance was not reached after adjusting for individual socio-demographic factors (9). Conversely, a German cohort study with a 9-year follow-up (n = 16,008; mean age: 53 years; 50.4% women) found no significant association between walkability and change in diabetes prevalence (12).

Similar results were observed for the relationship between green space/neighborhood aesthetics and risk of diabetes onset. Green space quantity is usually assessed within a radius of 1-3 km surrounding each participant’s home. In the study by Astell-Burt et al. (13) including 267,072 Australians (age > 45 years), residents in areas with 20% or less green space showed a higher rate of T2DM (9.1%) than those living in neighborhoods with 40% or more green space (8%). Furthermore, a study conducted in the UK on 10,746 adult participants (mean age: 59 years; 47% women; 21% Non-White ethnicity) found a negative correlation between green space and T2DM prevalence. Compared to the lowest quartile, diabetes ORs for increasing quartiles of green space were 0.97 (95% CI: 0.80 to 1.17), 0.78 (95% CI: 0.62 to 0.98) and 0.67 (95% CI: 0.49 to 0.93) respectively, after adjusting for social deprivation, urban status and individual-level covariates (14). Analogous findings were reported by Dalton et al. (15): 23,865 residents (mean age: 59 years; 55.1% men) in areas in the highest quartile of green space showed a 19% lower risk of developing diabetes than other quartiles, even after adjustment for demographic factors and SES. Interestingly, air pollution (i.e., high levels of NO2, PM2.5 and PM10) was associated with an increased risk of T2DM in the city of Leicester, UK (16).

### Neighborhood food environment

A research body of limited amplitude has investigated the role of neighborhood food store distribution, with emphasis on the density of food outlets selling unhealthy foods in residential areas. A large longitudinal cohort study conducted on 4,100,650 US veterans without T2DM (mean age: 59.4 years; 92.2% males; 76.3% Non-Hispanic White ethnicity) investigated diabetes incidence rate over a follow-up period of 5.5 person-years, taking into account variation in neighborhood food environment. A positive, moderate association was observed between fast-food density and T2DM risk, whereas supermarket density was associated with a lower T2DM risk only in suburban and rural contexts (17). In Mezuk et al. (18) a large cohort of 4,718,583 adult Swedes (min-max age= 35-80 years) was prospectively followed from 2005 to 2010: neighborhoods with ≥30% unhealthy food stores had the highest T2DM rate. Moreover, participants who moved to worse food environments showed increased diabetes risk. Similar observations revealed that a healthy food environment, combined with neighborhood physical activity resources, diminished the number of T2DM diagnosis among 5,124 US participants (mean age: 60.7 years; 53.6% women; 57.7% Non-White ethnicity) over a 8.9-year follow-up (19).

## Glycemic control and neighborhood characteristics

Neighborhood characteristics have been found to affect glycated hemoglobin (HbA1c) levels in different ways. A cross-sectional study (20) on 615 patients with T2DM (age > 18; 61.6% men; 65.7% Non-Hispanic Black ethnicity) showed that neighborhood aesthetics had a direct effect and access to healthy foods an indirect effect (i.e., mediated by self-care behaviors) on HbA1c % (β = 0.12, z = 2.19, p = 0.03 and β = - 0.17, z = -2.95, p = 0.003 respectively).

However, a subsequent analysis on the same sample (21) did not observe a significant effect of built environment features (namely neighborhood characteristics, neighborhood participation index and neighborhood problems index) on glycemic homeostasis in the fully-adjusted hierarchical model, whereas psychosocial factors (i.e., self-efficacy and social support) and comorbidities retained significance. Another cross-sectional study (22) on 424 TD2M patients (mean age: 60.5 ± 11.5 years; 52.8% women; 63.9% White ethnicity) reported that HbA1c % values were positively associated with higher scores of neighborhood social disorganization (B = 0.47, p = 0.003), a composite index including economic disadvantage, residential instability and ethnic heterogeneity. This effect was still significant (B = 0.39, p = 0.01) after controlling for demographic (race, age, sex, educational level) and psychosocial/clinical covariates (disease duration, diabetes distress, diabetes empowerment, selfcare, comorbidities). In a study conducted between 2013 and 2014 and stemming from the Heart Healthy Hoods project (23), which involved 269,942 electronic health records (EHR) of people aged ≥ 40 years living in Madrid (median age: 56.5 years, IQR age: 47.4- 69.8; 54.9% women), a composite neighborhood socio-economic status (SES) index was created (combining education, wealth, occupation and living conditions) and its association with HbA1c % levels among patients with T2DM was assessed. Using low SES neighborhoods as the reference group, and controlling for age and sex in the regression analysis, medium and high SES neighborhoods displayed decreased HbA1c % mean levels (mean change: -0.05, 95% CI: -0.01 to -0.10 and -0.11, 95% CI: -0.06 to -0.15, respectively) and a reduced prevalence ratio (PR) of uncontrolled diabetes (PR=0.95, 95% CI: 0.91 to 0.99 and PR= 0.91, 95% CI: 0.87 to 0.95, respectively). Within the Heart Healthy Hoods project, a sequential study (24) on 113,265 patients affected by T2DM (mean age: 62.7 ± 8.8 years; 56.8% men) confirmed a significant effect of neighborhood SES on glycemic control target (HbA1c % < 7.0%): after adjusting by sex and age, the SES index 5^th^ quintile (the most deprived) displayed a higher risk of uncontrolled diabetes (OR= 1.20, 95% CI: 1.10 to 1.32) than the reference group (SES index 1^st^ quintile). In a retrospective, longitudinal study (2007–2013) on 182,756 adults with diabetes living in New York City (mean age= 64 years; 47.4% White ethnicity) (25), a residential composite score (reflecting neighborhood socioeconomic status, food environment and aesthetics) was computed, with residential areas categorized into quintiles (1^st^ quintile= least advantaged, reference group; 5^th^ quintile= most advantaged) and incorporated into a multilevel model. Living in a 5^th^ quintile area was associated with a higher probability of reaching HbA1c % < 7% (OR= 2.59, CI 2.43 to 2.77), a shorter median time to reach glycemic control (median time 9.9 vs. 11.5 months; HR 1.14, CI 1.12 to 1.16) and lower HbA1c % levels (mean difference= - 0.44%). Finally, moving from the 1^st^ to the 5^th^ quintile residential areas was associated with better glycemic control (mean HbA1c % reduction: 0.40%, 95% CI 0.22 to 0.55), while the opposite was evident for those moving from the more advantaged to less advantaged areas (mean HbA1c % rise = 0.33%, 95% CI: 0.24 to 0.44).

Another retrospective longitudinal study on 2,662 patients (mean age: 69.3 ± 9.13 years; 55.3% women; 65.5% Non-Hispanic White ethnicity) (26) with self-reported diabetes investigated the role of four neighborhood factors (i.e., social cohesion, social participation, physical disorder, and perceived everyday discrimination) on glycemic control over time. In the unadjusted model, only social cohesion was significantly associated with HbA1c values (β = -0.05, 95% CI -0.10 to -0.001) among neighborhood factors; however, this relationship became non-significant after adjusting for demographic, psychosocial and financial factors.

A third retrospective longitudinal study (27) on EHR data of 15,308 patients affected by T2DM (mean age = 57.8 ± 11.9 years; 45.8% women; 96.4% Non-Hispanic White ethnicity) assessed the relationship between four community factors (namely: community socioeconomic deprivation, CSD; food availability; fitness and recreational assets; utilitarian physical activity favorability), HbA1c levels and pharmacological therapy intensification (TI), after stratifying by community type (townships, boroughs and city census tracts). Over a 6-month period, the mean HbA1c reduction was 0.07% lower in townships in the 3^rd^ CSD quartile vs. 1^st^ CSD quartile and 0.10% higher in townships in the 4^th^ food availability quartile (i.e., best food availability) vs. 1^st^ food availability quartile (i.e., worst food availability). In townships and boroughs, TI was associated with a smaller 6-month HbA1c reduction in the 4^th^ vs. 1^th^ CSD quartile, which was confirmed in the 24-month analysis only for census tracts. Finally, one prospective study (28) investigated the role of neighborhood walkability on 1-year glycemic control in a cohort of 1,230 patients with T2DM (mean age: 68.9 ± 9.0 years; 62.6% men) of the Diabetes Care System Cohort. Neither objective walkability (based on geographic information system) nor subjective walkability (questionnaire-based) showed a significant main association with changes in HbA1c and fasting glycemia; likewise, after adjusting for the mediator (total physical activity or moderate-vigorous physical activity, in turn) no direct effect was apparent.

## Access and adherence to therapy, access to healthcare services and other health outcomes

A number of studies investigated to which extent neighborhood characteristics affect self-care behaviors in the context of different diseases, showing a relevant impact on psycho-physical well-being. Concerning the relationship between environmental factors and diabetes, some authors focused on the interference of community and neighborhood on healthcare access and process (e.g., medication adherence).

A study on a cohort of 615 adults with T2DM (29) (South-Eastern US, one third White ethnicity, approximately two third were men), observed significant associations between neighborhood activities and some self-care behaviors, such as exercise (β= - 0.104), diet (β= - 0.072) and foot care (β= - 0.114). Exercise was also negatively associated with walking environment (β= -0.040), whereas medication adherence was negatively associated with food insecurity (β = -0.147). Based on data from 179 patients enrolled in a prospective randomized controlled trial (30) (of whom two third were women and one third was White), the adherence to oral hypoglycemic agents was examined according with some neighborhood-related characteristics, namely: social affluence, residential stability 12.91, 95% CI: 2.20-75.80). In addition and neighborhood advantage. Patients living in a neighborhood with all the above-mentioned factors had a better adherent pattern than residents living in neighborhoods with low indicators (adjusted OR: 12.91, 95% CI: 2.20-75.80). In addition to adherence patterns, some authors investigated the access to different medication categories based on neighborhood features. In a large cohort of 1,203,317 Australian individuals with T2DM, Morton et al. examined diabetes medication dispensing based on remoteness.

Glucagon-like peptide-1 receptor agonists (GLP-1RAs) were less prescribed in patients living in regional areas compared to their counterparts living in major cities, though this difference was blunted over time (31). When considering remote areas, a delay was observed in the access to any new glucose- lowering drug, requiring from five to seven years since medication release (for Dipeptidyl peptidase-4 inhibitors, DPP4i) and GLP-1RAs, respectively) to reduce the magnitude of the difference compared to major cities. As for SGLT2 inhibitors, patients were less likely to receive those medications in remote areas compared to major cities. Kowitt et al. (22) found that among 450 patients with T2DM, living in neighborhoods with high economic disadvantage was positively associated with the use of acute/emergency health care services, compared to the medium economic disadvantage as the reference group (β = 0.60, 95% CI: 0.10 to 1.09). However, when other neighborhood indicators of social disorganization were taken into account and considered as a single composite measure (namely, neighborhood economic disadvantage, residential instability, as ethnic heterogeneity), no association emerged with self-reported use of acute/emergency health care services.

A Canadian study examined non-drug related healthcare costs among 698,112 adults with diabetes, revealing that individuals in the young age class (20-39 years) exhibited an increase of costs up to 41.3% (32) in the lowest quintile of neighborhood socioeconomic status compared to Ontarians with diabetes in the highest quintile.

In another Canadian study (33) involving a cohort of immigrants and long-term residents with diabetes (n = 175,414), individuals living in urban areas had a significantly reduced risk for cardiovascular events or death (fully adjusted HR: 0.85, 95% CI: 0.76-0.95), underscoring the relevant impact of the neighborhood of settlement.

According to data from the 2014 Health Center Patient Survey (34), unstable housing (37% out of 1,087 participants) was related to a five-fold increase of the risk of diabetes-related emergency department and hospital use in the past year (OR: 5.17, 95% CI: 2.08 to 12.87) compared to stable housing, after adjustment for potential confounders. A whole-population longitudinal study (35) conducted in England investigated the changes in the slope indices of inequality (SIIs) between neighborhoods of least versus greatest deprivation (for a total of 32,482 neighborhoods). From 2004/2005 to 2011/2012 emergency hospitalizations for diabetes increased especially in neighborhoods of greater deprivation (SII change: +19.59 admissions for diabetes per 100 000, 95% CI: 16.00 to 23.17). Conversely diabetes-related amenable mortality decreased at a faster rate in neighborhoods of greater deprivation, with the SII reduced by 2.68 (95% CI: 1.93 to 3.43).

## Discussion

**Figure 1:**
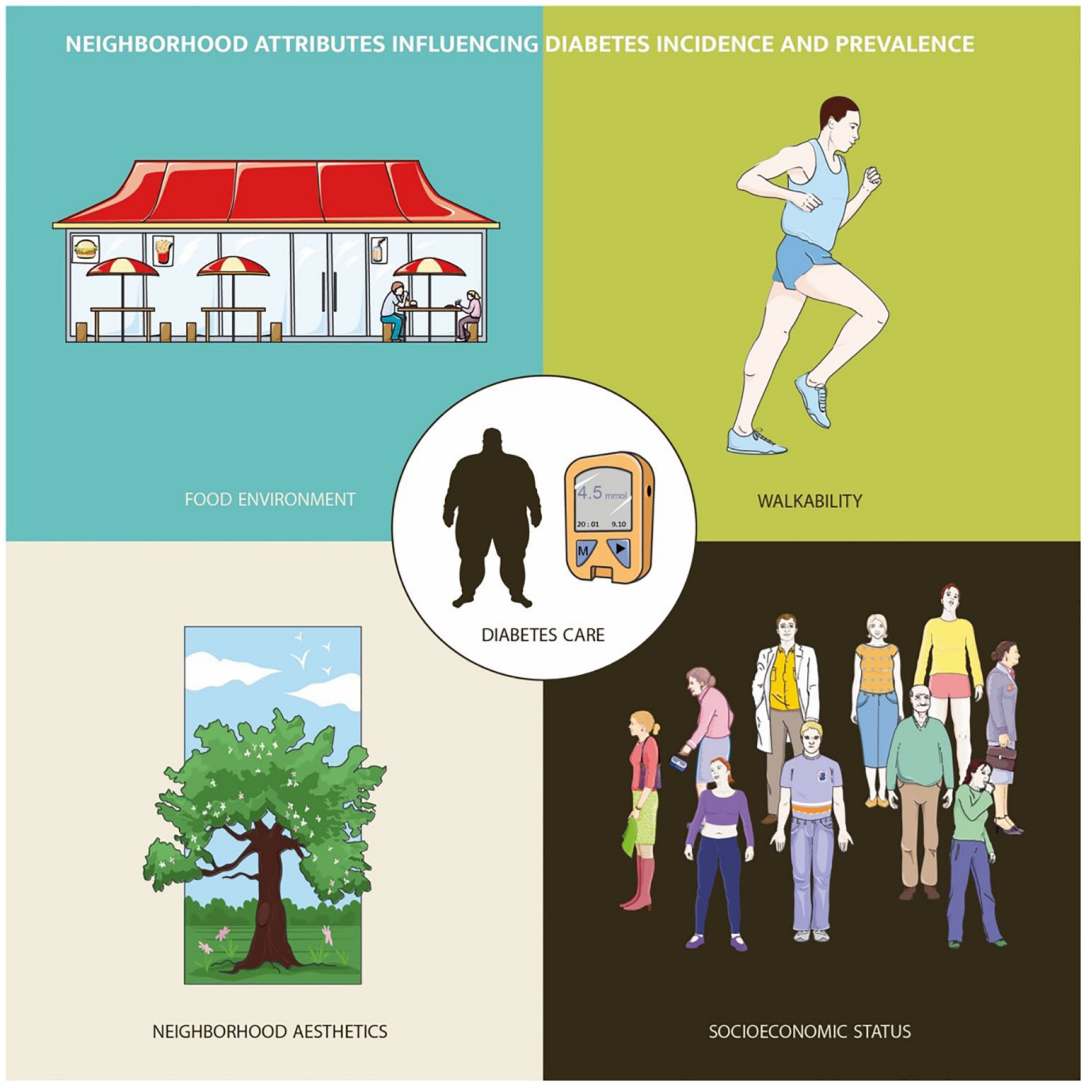
Neighborhood attributes influencing diabetes epidemiology and care.

Diabetes-related dimensions – from disease onset to access to medical therapy and glycemic control – have been thoroughly studied during the last decade; in this context, different neighborhood attributes can modulate diabetes epidemiology and care (**Figure 1**). For instance, walkability, greenspace presence and air quality in neighborhoods were correlated with reduced diabetes incidence and prevalence; as a matter of fact, factors other than neighborhood environmental (including food and beverage retail composition) and geographical characteristics may have mediated the relationship between areas of residence and diabetes mellitus risk.

Of note, at times the effects of residential socio-economic disparities were no longer apparent after adjusting for individual characteristics. For example, using glycemic control as the outcome measure, the included studies were concordant on the role of specific residential socioeconomic, food environment and aesthetic characteristics on HbA1c % levels, both at baseline and prospectively. Nonetheless, when considering composite neighborhood scores and after controlling for baseline covariates, this relationship became non-significant in some models. Continuously living in or moving to a more advantaged neighborhood was also associated with a higher probability of reaching glycemic control and reduced time-to-event, in comparison with disadvantaged areas. However, neighborhood walkability and subjective walkability were neither directly nor indirectly associated with glycated hemoglobin values, whereas the availability of fitness assets was positively associated with glycemic control in one work. It is worth highlighting that this lack of significance may be partly explained by the known inaccuracy of self-reported questionnaires regarding individual physical activity levels (36) and by the lack of variability in objective neighborhood walkability, as the authors of the paper suggested (28). Indeed, a homogenous level of exposure to this (putative) risk factor would hinder the statistical power of observational studies to find a significant association (37). Analogous observations underscored the remarkable role of neighborhood deprivation on access to medications, novel medication dispensing, adherence to antidiabetic therapy, and access to healthcare services related to T2DM. On the other hand, medication adherence was negatively associated with food insecurity, indicating that cultural factors (neighborhood-related and/or neighborhood-mediated) can exacerbate the connection. The so-called “causes of incidence” (based on Geoffrey Rose’s theory on the differences in individual and population causes of diseases) stem from the population’s social, cultural, economic and political contexts that make environments detrimental for the promotion of the efficacy of prevention strategies (37). In conclusion, it is crucial that policymakers develop evidence-based policies at national and regional levels based on effective multidimensional treatments. The latter should not solely rely on tackling individual risk factors, which may be particularly inefficient among the most deprived (38), but on population-level interventions in order to display a true impact on diabetes inequalities.

## Author contributions

FF: Investigation, Methodology, Writing – original draft. LM: Data curation, Software, Visualization, Writing – original draft. AP: Supervision, Writing – review & editing, Validation. LD: Supervision, Writing – review & editing, Resources, Validation. EP: Conceptualization, Writing – original draft, Methodology, Supervision, Validation.
